# Disclosing Trends on C(1s) Core-Level Binding Energies of MXene Flakes: Metal, Size and Edge Effects

**DOI:** 10.1021/acs.jpcc.6c03476

**Published:** 2026-08-10

**Authors:** Miquel Allés, Sergi Vela, Carmen Sousa, Francesc Illas

**Affiliations:** † Departament de Ciència de Materials i Química Física & Institut de Química Teòrica i Computacional (IQTCUB), 16724 Universitat de Barcelona, c/Martí i Franquès 1-11, Barcelona 08028, Spain; ‡ Institut de Química Avançada de Catalunya (IQAC−CSIC), c/Jordi Girona 18-26, Barcelona 08028, Spain

## Abstract

The C­(1s) core-level binding energies (CLBE) of realistic MXene finite systems were investigated using density functional theory (DFT). The ΔSCF approach was employed to explicitly account for final state effects. MXene flakes with M_2_CT_*x*_ stoichiometry (M = Sc, Y, Ti, Zr, Hf, V, Nb, and Ta, where T_*x*_ denotes surface termination), consisting of 18 stoichiometric units, were constructed in both pristine (M_2_C)_18_ and O-functionalized (M_2_CO_2_)_18_ forms. CLBEs were computed for all C atoms in each flake using the PBE functional, which was found to correlate linearly with results from the more accurate hybrid PBE0 functional. An increase in CLBEs is observed across periods, driven by decreasing atomic radii and increasing metal electronegativity, whereas trends along groups follow a behavior similar to that of first ionization potentials. O-functionalized flakes exhibit higher CLBEs due to the electronegative effect and the structural distortion caused by the O termination. This enables a clear distinction between pristine and O-terminated materials in XPS spectra. In addition, the flake size effect on central carbons in (Ti_2_C)_*n*_ and (Ti_2_CO_2_)_*n*_ flakes, with n up to 216, is negligible, supporting the extrapolation of the present results for larger systems. Overall, the C­(1s) XPS spectra of MXene flakes are expected to be centered around the CLBE of the central C atom, with peak widths determined by the difference between the maximum and minimum CLBE values.

## Graphical abstract

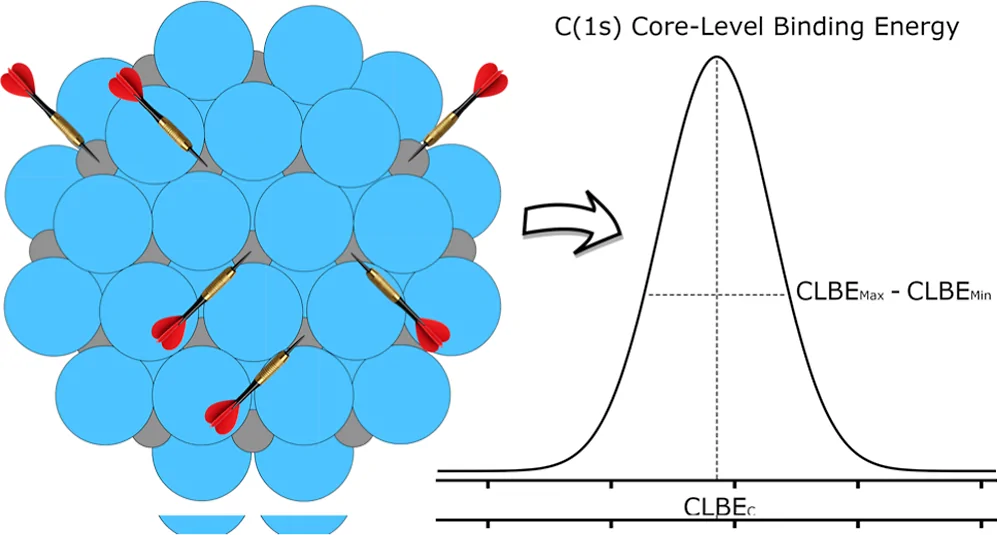

## 1 Introduction

MXenes are a class of 2D materials first synthesized in 2011 by Naguib and co-workers.^1^ Their name, reminiscent of graphene, highlights their 2D nature and derives from their chemical composition. MXenes consist of transition-metal carbides and nitrides, where M denotes the transition metal (typically from groups III to VI), and X represents either C or N.^2^ Their synthesis involves the exfoliation of a MAX phase precursor, a 3D material in which MXene layers are interleaved with intermediate A layers (usually an element from Groups XIII or XIV).^3,4^ In general, MAX phases follow the general M_*n*+1_AX_*n*_ chemical formula, where *n* ranges from 1 to 4 and indicates the number of X layers.^5^ Due to the exfoliation process, MXenes are typically obtained with a variety of functional groups attached to their outermost layers. For this reason, the notation M_n+1_X_n_T_*x*_ is commonly used, where T_*x*_ denotes the surface terminations composed of various functional groups introduced during the synthesis. The most common terminations include –F, –O, –OH, and –H.^6^ However, more recent studies have reported MXenes with alternative surface terminations, such as –S, –Se, –Te, –NH, –Cl, and -Br, demonstrating the increasing tunability of MXene surface chemistry.^7,8^ Importantly, pristine MXenes without surface terminations have also been reported.^9–11^

From the structural point of view, MXenes usually adopt a hexagonal lattice with ABC stacking, inherited from the MAX phase.^2,10,12^ However, theoretical studies have predicted that ABA stacking can be energetically favorable in certain cases,^7,13–15^ with implications for their observable properties.^16,17^ MXenes containing early transition metals from Groups III to V tend to prefer ABC stacking, whereas those based on Group VI transition metals favor an ABA arrangement.^14^ Upon functionalization with oxygen atoms, ABA stacking structures tend to accommodate the O atoms at H_X_ (or *hcp*) sites (see Figure 1).^14^ In contrast, for ABC-stacked structures, two different configurations can emerge; in Group IV and V MXenes, O atoms preferentially occupy H_M_ (or *fcc*) sites (see Figure 1), whereas Group III MXenes adopt a Janus-type structure, with adsorption at H_X_ sites on one surface and H_M_ sites on the other.^14^

**Figure 1:**
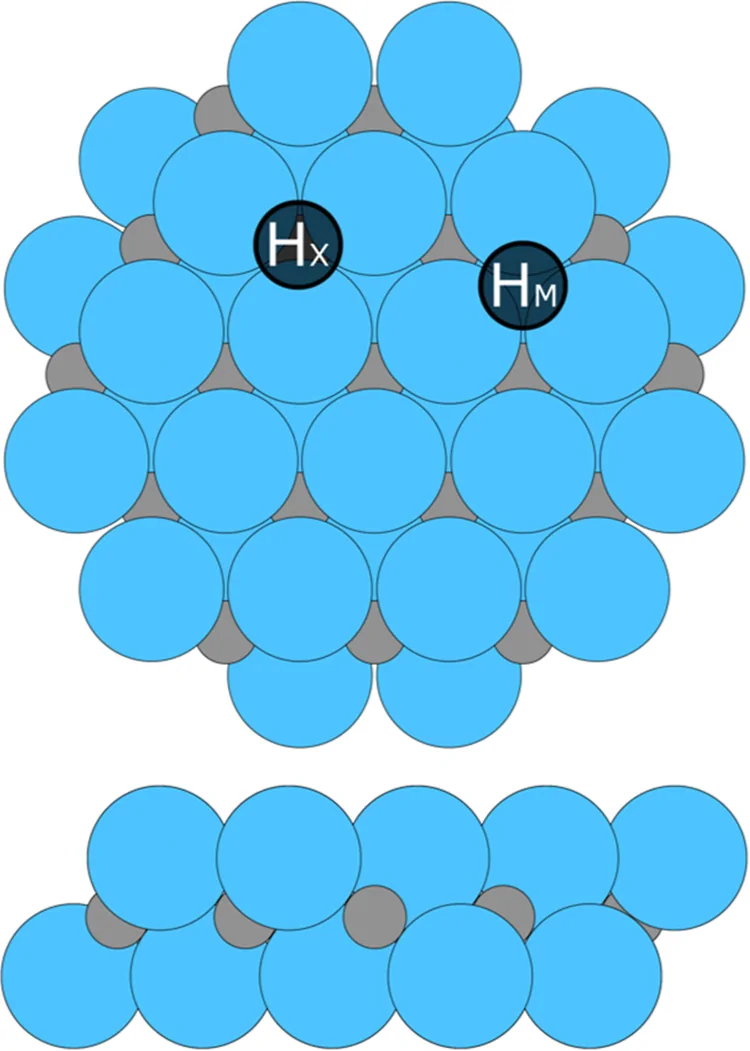
Atomic structure of the investigated (M_2_C)_18_ MXenes flakes. H_M_ and H_X_ sites for oxygen adsorption on (M_2_CO_2_)_18_ flakes are shown in black. Blue and gray spheres refer to M and C atoms, respectively.

Experimentally, MXenes are challenging to characterize. The etching process used during their synthesis often makes it difficult to determine whether the resulting MXenes are functionalized, with possible mixed functional groups as well.^18,19^ To address these challenges, a variety of experimental techniques are employed, among which X-ray photoelectron spectroscopy (XPS) is the most widely used. Since its development in the late 1960s, following the comprehensive work of Siegbahn and co-workers,^20,21^ and the construction of the first XPS instrument in 1969, this technique has become a cornerstone of materials characterization. XPS is based on the Einstein interpretation of the photoelectric effect. In this method, the binding energy of a core electron in a molecule can be determined from the difference between the energy of the incident X-ray photon and the kinetic energy of the emitted photoelectron. This energy, commonly referred to as the core-level binding energy (CLBE), is characteristic of the ionized atom and provides valuable information about its chemical state and local atomic environment. In the case of solids, the surface work function also needs to be considered.

To distinguish chemical environments within a system, it is sufficient to compare the CLBE values associated with the same element at different atomic sites (see Figure S1 in the Supporting Information). However, when comparing different materials, it is necessary to define a common CLBE reference, from which relative shifts (ΔCLBEs) can be determined. Overall, the CLBE values assigned to specific elements, together with the intensities of their corresponding peaks in the spectrum, are related to the concentrations of the constituent species and thus provide both qualitative and quantitative analytical information. In contrast, ΔCLBE values offer insight into the nature of the chemical bonding and the local electronic environment of the ionized atom.^22–25^

From a computational perspective, CLBE values are usually estimated via the so-called ΔSCF approach, first introduced in the seminal work of Bagus in 1965.^26^ This method relies on the total energy difference obtained from two self-consistent field (SCF) calculations: (i) the ground state of the system of interest, and (ii) a core-ionized state in which one electron is removed from a selected core orbital, which for first-period elements typically involves the 1s orbital. For molecular systems, the energy difference between these two states directly yields the CLBE of the system and can therefore be compared with experimental XPS measurements. The reliability of the ΔSCF approach in predicting ΔCLBE values has been established for a large data set of gas-phase molecules.^27^ One advantage of studying isolated molecules is that the surface (or spectrometer) work function does not need to be considered, in contrast to condensed matter systems. This simplifies the comparison between computational and experimental CLBEs.^22^ Analyses of various Density Functional Theory (DFT) methods indicate that the generalized gradient approach (GGA) based PBE functional^28^ generally provides reliable and sufficiently accurate CLBEs. Nevertheless, hybrid functionals such as PBE0 ^29,30^ and HSE06,^31^ as well as meta-GGA functionals such as TPSS,^32^ tend to perform slightly better, yielding smaller absolute errors.^27^

In the case of MXenes, XPS data remain relatively scarce for computational studies. For MXenes with M_2_C and M_2_CO_2_ stoichiometries, a relatively recent study focusing on the C­(1s) and O­(1s) CLBEs reported a good agreement between calculated and experimental ΔCLBE results,^33^ offering interesting trends as well. For instance, the computational analysis revealed a strong correlation between ΔCLBEs and the atomic charges of C and O in the neutral system. More recently, a combined experimental and computational study reported CLBEs for C­(1s), O­(1s), Ti­(2p), and F­(1s) in Ti_3_C_2_T_*x*_ MXenes.^34^ In these two previous works, the computational analysis was performed using periodic models to represent the MXene materials. However, one must point out that the estimate of CLBEs from periodic models inherently involve several approximations, since the ΔSCF approach cannot be directly applied to 3D periodic systems as the vacuum level is not well-defined. This is not the case of 2D periodic systems like MXenes as one can introduce a large enough vacuum region in the unit cell. Nevertheless, there are additional limitations arising from describing the core-ionized final state within a charged unit cell. Charge neutrality can be restored using approximate correction schemes, or alternatively by employing the well-stablished Janak-Slater method, which treats electron removal through fractional electron occupations. Despite their limitations in accurately predict absolute values of CLBEs, these methods are generally able to reproduce ΔCLBEs with good accuracy. Moreover, because of the periodic symmetry inherent to these idealized models, they usually contain a single type of C atom only, whereas real samples consist of finite flakes with distorted geometries and/or defects, especially near the flake edges, giving rise to multiple distinct chemical environments for C atoms. Thus, important questions emerge related to whether these different C atoms can be distinguished in XPS measurements, and, if so, whether they exhibit ΔCLBE values that depend on the flake size.

These are precisely the questions addressed in the present work. To this end, the ΔSCF approach within DFT based methods has been used to determine the C­(1s) CLBE of a series of MXene flakes whose structural and electronic properties were analyzed in recent studies.^35,36^ In particular, we consider the most stable pristine (M_2_C)_18_ and O-terminated (M_2_CO_2_)_18_ flakes with ABC stacking and M = Sc, Y, Ti, Zr, Hf, V, Nb, and Ta. The selected metals correspond to groups 3, 4, and 5 of the periodic table, and cover the 3d, 4d, and 5d transition series. This choice allows the trends in CLBE to be analyzed across both periods and groups, providing a broad and systematic picture of the transition metal effect on the CLBE. Besides, this structure selection allows the study to focus on the effects of the metal and functionalization, without introducing stacking-dependent factors. The use of finite models, as opposed to slab models, allows us to avoid the Janak-Slater approximations in the description of the final ionized state, thereby providing a more realistic representation of the system. The outcome of this work reveals a metal-dependent trend in the C­(1s) CLBEs, evolving differently across periods and groups. Surface O functionalization leads to an increase in CLBEs for all cases. The calculated shifts are in line with previous studies using periodic models, differing only slightly in magnitude. Overall, this work provides a comprehensive picture of C­(1s) CLBEs in (M_2_C)_18_ and (M_2_CO_2_)_18_ flakes and provides estimations for the position and shape of the corresponding experimental XPS spectra. Furthermore, the analysis of the flake size effects indicates that CLBEs do not significantly change for central C atoms in Ti-based flakes, suggesting that extrapolation to larger systems may be feasible.

## 2 Models and Computational Details

In the present work, ΔSCF calculations are used to predict the C­(1s) CLBE of all carbon atoms in 16 representative MXene flakes, including eight pristine (M_2_C)_18_, and eight O-terminated (M_2_CO_2_)_18_ models. All structures correspond to MXenes with M_2_C stoichiometry and M = Sc, Y, Ti, Zr, Hf, V, Nb, and Ta, for which ABC stacking is the most stable configuration in both pristine and O-functionalized forms. These structures were selected to study the functionalization effect of the most common O termination, a more systematic study involving other terminations is highly desirable and will be considered in the future. The geometries of the flakes were defined as reported in a previous study, in which the structures were constructed considering the lowest energy surface faces and subsequently fully optimized using DFT calculations.^36^ All pristine flakes share the same structure, as shown in Figure 1. In contrast, two configurations arise for O-terminated flakes; with O atoms adsorbed on H_M_ sites in group IV and V metal-based MXenes, whereas in Sc- and Y-based flakes, O atoms occupy H_M_ and H_X_ sites on opposite surfaces. Exceptionally, for (Ti_2_C)_*n*_ and (Ti_2_CO_2_)_*n*_ MXenes, seven flake sizes (*n* = 18, 36, 60, 90, 126, 168, and 216) were modeled to evaluate the size effects on the C­(1s) CLBE, with structures taken from previous work.^36^

For the calculation of CLBEs, two approaches are commonly employed in the literature, often referred to as the “initial state” and “final state” methods. Both rely on the total energy difference between two SCF calculations (ΔSCF), where the CLBE is obtained as the difference between the total energy of the ground state and that of a core-ionized state in which one electron has been removed from a core orbital. The distinction between these two approaches lies in how the ionized state is described. In the initial state approximation, only the removal of a core electron is considered, neglecting the relaxation of the electronic structure in response to the core hole. At the Hartree–Fock level, the Koopmans’ theorem states that the ionization energy corresponds to the negative of the energy of the corresponding core orbital in the ground state.^23–25^ Although this relationship does not strictly hold within DFT, initial state CLBEs can still be rigorously defined within this framework.^37^ In contrast, the final state approach explicitly accounts for the relaxation of the electron density following the creation of a core hole. This relaxation energy can be substantial, typically ranging from 10 to 30 eV for 1s core electrons. Nevertheless, these large relaxation contributions are essentially local in nature and, although they significantly affect the absolute CLBE values, their impact on core-level shifts (ΔCLBE) is considerably smaller, as these are mainly governed by initial state effects.^23–25^ In this context, distinguishing between initial and final state effects is essential. In XPS, information about a material in its neutral state is inferred from measurements performed on the ionized system. Therefore, it is crucial to assess whether an observed core-level shift is already present in the neutral sample or arises primarily from relaxation effects in the ionized state. Such information can only be accessed through theoretical calculations.

In this work, we focus on the ΔSCF approach within the DFT framework, which explicitly includes final state effects. A performance comparison of initial/final state approaches can be found in Section S1 of the Supporting Information, where we demonstrate that final state methods yield more accurate CLBE values. Technically, final state calculations are performed by converging the orbitals of the ionized system using the Maximum Overlap Method (MOM), rather than energy minimization criteria based on the Aufbau principle. For each structure, the different CLBEs have been obtained as

1 $$CLBE=E_{n-1}-E_{n}$$

where *E*_*n*_ is the energy of the ground state of a given MXene flake, and *E*_*n*–1_ is the energy of the same system with one electron removed from the chosen C­(1s) core orbital. Within this approach, all CLBEs are referenced to the vacuum level, enabling straightforward comparison between different MXenes.

All density functional theory (DFT) calculations were carried out using the Fritz-Haber Institute Ab-Initio Materials Simulation (FHI-AIMS) software.^38^ All electrons were explicitly treated using numeric atom-centered orbitals (NAO) with a light tier-1 basis set.^39^ Relativistic effects were included through the zeroth-order regular approximation (ZORA), originally developed by Chang et al.,^40^ and later popularized by van Lenthe et al.^41^ through its implementation in the ADF code. For most calculations, the Perdew–Burke–Ernzerhof (PBE) functional^28^ was used to account for the contributions of exchange, correlation and kinetic energy of interacting electrons. Dispersion correction schemes were not employed, since we do not expect van der Waals interactions to be relevant for the computation of CLBE in our 0D MXene flake models. For the structure optimization, the atomic forces were converged below 10^–2^ eV·Å^–1^ with the Broyden–Fletcher–Goldfarb–Shanno (BFGS) optimization algorithm, and the electronic structure was converged below 10^–5^ eV using a Gaussian broadening of 0.01 eV. For the ΔSCF calculations, in some cases the electronic converged threshold was relaxed to 10^–3^ eV due to convergence difficulties. Additionally, for comparative purposes, the more accurate hybrid PBE0 functional^30^ was used to compute C­(1s) CLBE values for the central C atom of each MXene.

## 3 Results and Discussion

To assess the accuracy of the CLBE values computed with the present computational setup, an initial benchmark was performed. The C­(1s) CLBEs of ethyl trifluoroacetate, also known as ESCA molecule and commonly used as a reference system for XPS validation,^42^ were evaluated using different basis sets and exchange–correlation functionals (i.e., PBE and PBE0). The results show only minor differences upon changing the basis set, with variations below 0.1 eV, but a significant improvement, up to 0.5 eV, when using the hybrid PBE0 functional (see Table S1 in the Supporting Information).^30^ For this reason, the C­(1s) CLBEs of the central C atoms in the studied MXene flakes were computed using PBE and PBE0 functionals.

For the set of explored (M_2_C)_18_ flakes, Figure 2 shows that PBE and PBE0 computed CLBEs correlate linearly, indicating that changing the functional results in an approximately constant shift in the calculated CLBEs. For pristine flakes, this correlation is strong (*R* ^2^ = 0.993), with PBE0 CLBE values lying between 0.07 and 0.37 eV higher than the corresponding PBE results. On the other hand, O-terminated flakes exhibit a slightly poorer correlation (*R* ^2^ = 0.881), indicating that the impact of the exchange–correlation functional is not uniform across all systems. For these cases, PBE0 CLBEs are generally 0.26–0.48 eV larger, with the exceptions of (Sc_2_CO_2_)_18_ and (V_2_CO_2_)_18_, which show differences of −0.25 and 1.10 eV, respectively. These outliers can be attributed to the different treatment of self-interaction error in the PBE and PBE0 functionals, which affects the 3d transition metal series, with the exception of Ti due to its more stable d^0^ electronic configuration.

**Figure 2:**
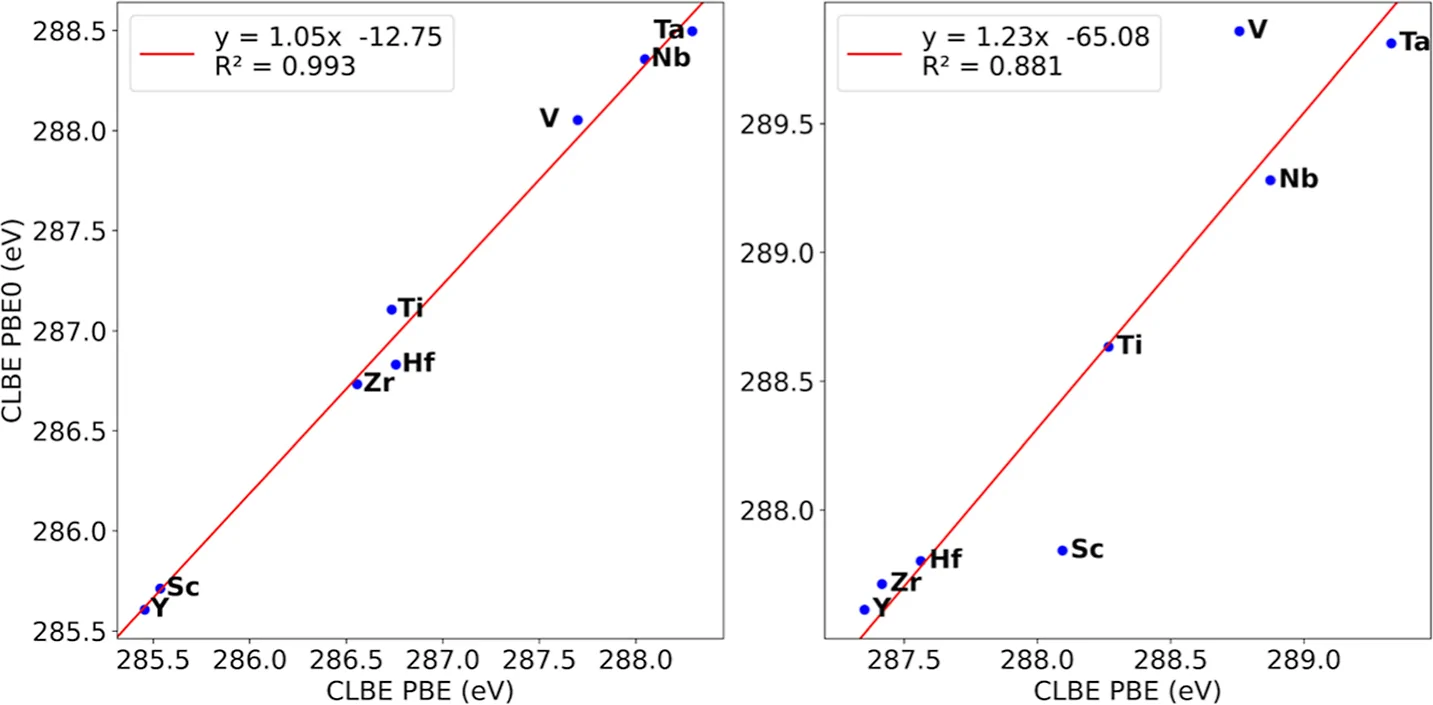
Comparison between ΔSCF CLBE values calculated with PBE0 and PBE functionals for the central C atoms of (M_2_C)_18_ (left) and (M_2_CO_2_)_18_ (right). The red line shows the results of a linear regression with the inset parameters.

Overall, PBE captures the trends in C­(1s) CLBE values for both (M_2_C)_18_ and (M_2_CO_2_)_18_ structures. Although some deviations are observed on O-functionalized structures, PBE is considered sufficiently accurate for a systematic study involving all C atoms of each flake, especially given its lower computational cost. In total, 288 C­(1s) CLBEs were obtained, encompassing 16 MXene flakes with 18 carbon atoms per flake. All structures exhibit ABC stacking, therefore, stacking-dependent effects are not accessible within the present study. For Sc- and Y-based systems, oxygen adsorption sites differ depending on the surface of the flake (H_M_ on one side and H_X_ on the other), whereas for the remaining metals, oxygen preferentially adsorbs at H_M_ sites, as already reported.^36^

### 3.1 CLBEs of (M_2_C)_18_ and (M_2_CO_2_)_18_ MXene Flakes

Each of the 18 C atoms in the MXene flakes leads to a distinct C­(1s) CLBE value. These values can vary substantially due to finite-size effects, such as structural distortions and presence of low-coordinated sites, which give rise to markedly different local environments and, consequently, to a distribution of CLBE values within each flake. Rather than discussing all individual values, three representative descriptors are considered: (i) the maximum CLBE value (CLBE_Max_), (ii) the minimum CLBE value (CLBE_Min_), and (iii) the CLBE value of the central carbon atom (CLBE_C_). CLBE_C_ is used to assess the type of carbon environment expected to dominate in larger flakes or periodic models, where finite-size effects are less pronounced.^33^ In turn, CLBE_Max_ and CLBE_Min_ define the full range of CLBE values and thus provide an estimate of the expected width of the bands in the experimental XPS spectrum. Overall, smaller flakes are expected to exhibit broader peaks, whereas increasing flake size leads to peak narrowing as the edge-to-central-atom ratio decreases. Interestingly, the C­(1s) feature in Ti_3_C_2_ is relatively broad, in agreement with present results.^43^ However, direct comparison with experiment is not straightforward because the present CLBE values are referenced to the vacuum level whereas experimental studies often adopt pragmatic energy referencing schemes to avoid challenging calibration procedures. For instance, one of the fitting procedures reported in ref 43 sets the C–Ti–F component peak at 460.2 eV and assigns the C­(1s) peak to approximately 282 eV. Although this differs substantially from the present calculations, the discrepancy is clearly influenced by the chosen reference energy. The same study also reports an alternative fit in which C­(1s)-related features span the 282–286 eV range. One interesting observation regarding the present results is that, except for the edge atoms, all C atoms within the M_2_C flakes experience very similar chemical environments. This finding calls into question the assignment of individual fitted C­(1s) peaks to distinct bonding configurations, such as Ti–C or C–C, since the calculated chemical environments do not support such a clear distinction.

Having stablished that direct comparison of absolute CLBEs values with experiment is not straight, we now examine the trends in calculated CLBEs in greater detail. Starting with pristine MXenes, an increase in CLBE_C_ is observed across periods (see Figure 3 and Table S2 in the Supporting Information). This trend is associated with the decreasing atomic radii and increasing electronegativity of transition metals, which leads to shorter C–M bond distances (see Figure S2). Both effects contribute to strengthening the C–M bonds, thereby increasing the energy required to remove an electron from the core orbitals. In contrast, this trend is not observed when comparing along groups, where a decrease in CLBE is found between the first and second transition metal series for Groups III and IV, while a constant increase is retained for Group V. This behavior is also reflected in the first ionization potentials (IP) of these metals (see Table S3 in the Supporting Information). Regarding the CLBE range (CLBE_Max_ – CLBE_Min_), it varies from 0.30 for (V_2_C)_18_ to 0.63 eV for (Ti_2_C)_18_, indicating that the theoretical XPS spectra of pristine MXenes are well-defined and exhibit relatively narrow peaks.

**Figure 3:**
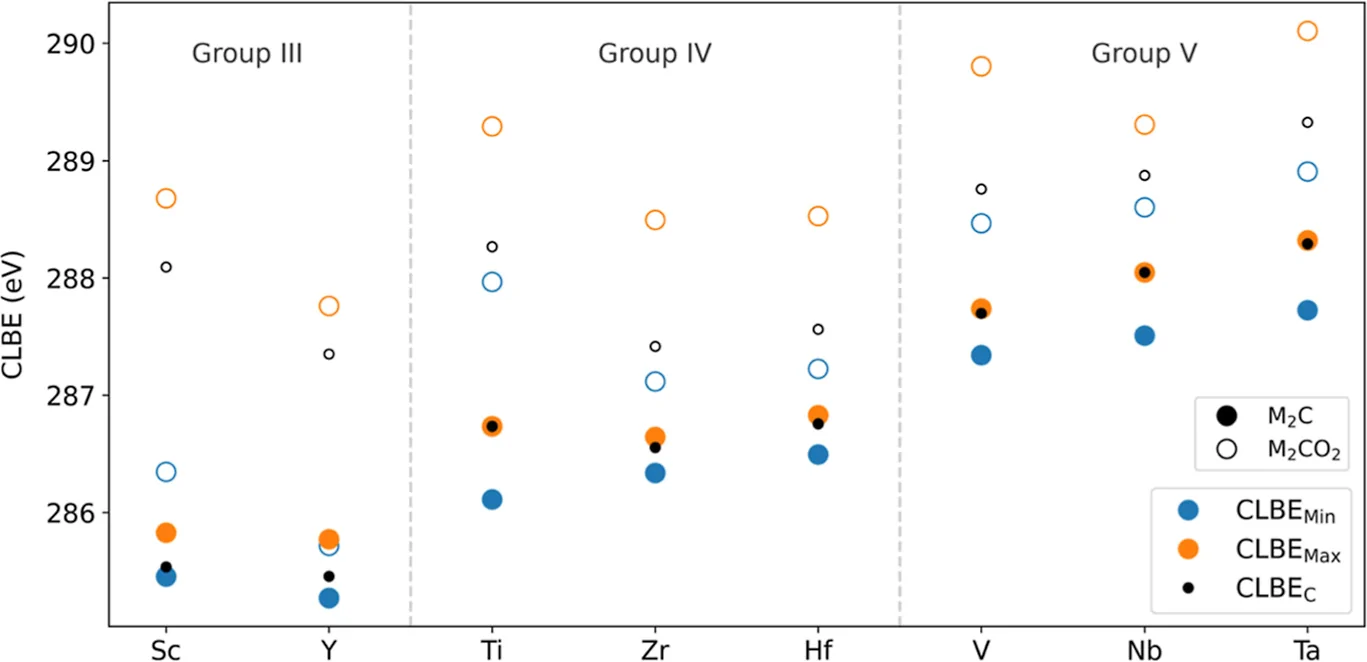
Calculated CLBE for all C atoms in (M_2_C)_18_ and (M_2_CO_2_)_18_ MXenes, ordered by groups according to their transition metal. The minimum and maximum values for each flake are shown in blue and orange, respectively, while the energy of the central carbon atom is shown in black. Filled and open circles correspond to (M_2_C)_18_ and (M_2_CO_2_)_18_ MXenes, respectively.

For the O-terminated (M_2_CO_2_)_18_ systems, the same general trend is observed, with CLBEs increasing across periods and decreasing along groups III and IV. In all cases, C­(1s) CLBEs are shifted to higher energies relative to the pristine counterparts due to the strong electronegative effect of oxygen, which withdraws electron density from both carbon and metal atoms (see Figure S3), thereby inducing a net positive charge on those atoms. Nevertheless, O-functionalized flakes exhibit structural distortions induced by the formation of M–O bonds. These distortions lead to a slight bending of the flakes and enhance the differentiation between C atoms compared to pristine structures. This effect is particularly pronounced in Janus-type flakes, i.e. (Sc_2_CO_2_)_18_ and (Y_2_CO_2_)_18_, which display the largest CLBE ranges (CLBE_Max_ – CLBE_Min_) of 2.33 and 2.04 eV, respectively. In contrast, for group IV and V flakes, the CLBEs range between 1.38 and 0.71 eV for (Zr_2_CO_2_)_18_ and (Nb_2_CO_2_)_18_, respectively.

Moreover, the oxygen adsorption sites (H_M_ or H_X_) influence the CLBE distribution. Sc- and Y-based flakes, in which oxygen adsorption occurs on H_X_ and H_M_ sites on opposite surfaces, exhibit CLBE_Min_ values that deviate significantly from CLBE_C_. In contrast, for Group IV and V metal-based flakes, where oxygen adsorption occurs exclusively at H_M_ sites, the largest deviation from CLBE_C_ is observed for CLBE_Max_. This behavior is associated with the H_X_ sites, where oxygen atoms are adsorbed directly above C atoms, enabling strong C–O interactions that induce a partial positive charge on carbons and consequently increase the corresponding CLBE values. Notably, CLBEs of the O-terminated systems are significantly higher than those of the pristine counterparts (see Table S2 in the Supporting Information). For the central C atom, shifts of approximately 2 eV are observed for Group III metals, 1.6 eV for Ti, and around 1 eV for the remaining metals, indicating that the presence of O terminations in MXene structures should be distinguishable in XPS experiments.

### 3.2 Size Effects on the CLBEs of (M_2_C)_*n*_ and (M_2_CO_2_)_*n*_ MXene Flake

CLBE values of a given atom are often difficult to compare directly with experimental or computational data reported in the literature due to differences in energy references used for instrument calibration, as well as to the various approximations adopted in theoretical studies. To bypass this problem, CLBE shifts (ΔCLBE) are usually defined by adopting a common reference (see Figure S1). In this work, to allow comparison between CLBE values obtained using flakes and periodic MXene models, all CLBE values are referenced to Ti-based MXenes. Thus, ΔCLBEs values for pristine MXenes are defined relative to Ti_2_C, while those for O-functionalized MXenes are referenced to Ti_2_CO_2_ (see Figure 4 and Table S4 in the Supporting Information). This convention is applied consistently to both the studied flakes and the reported periodic model data.^33^

**Figure 4:**
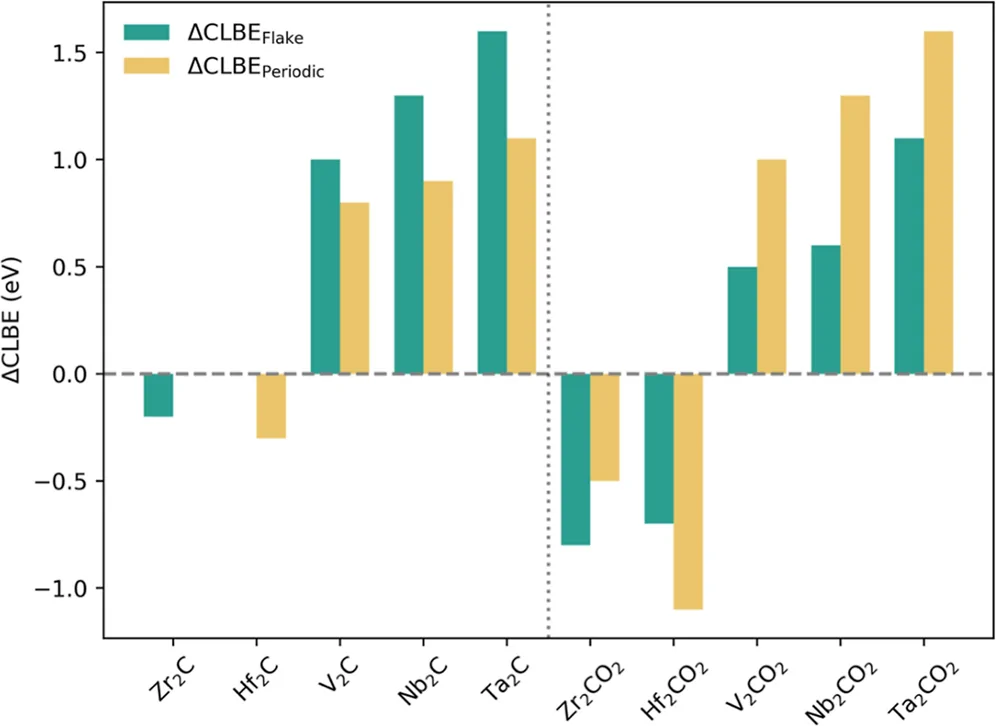
Core-level binding energy shifts (ΔCLBE) for the central C atom of MXene flakes (ΔCLBE_Flake_) and of MXene using a periodic model (ΔCLBE_Periodic_).^33^ Ti-based MXenes were used as a reference.

The central C­(1s) ΔCLBEs in pristine and O-terminated MXenes flakes and that from periodic models exhibit similar trends. However, for pristine flake models ΔCLBE values are larger than those predicted by the periodic model (ca. 0.5 eV), whereas for O-functionalized flakes the ΔCLBE values are smaller (ca. 0.7 eV) than those obtained from their periodic counterparts (see Figure 4). Only Zr_2_C and Hf_2_CO_2_ deviate from these trends. This different behavior between pristine and functionalized flakes is attributed to structural distortions present in terminated flakes which, by construction, are not found in the periodic models, unless very large cells are used.^33^ Pristine flakes exhibit nearly periodic-like structures with only minor distortions, whereas O-functionalized flakes adopt slightly distorted configurations, and therefore deviate more strongly from the periodic behavior.

Additionally, the evolution of the C­(1s) CLBEs with flake size was evaluated for (Ti_2_C)_*n*_ and (Ti_2_CO_2_)_*n*_ flakes with *n* = 18, 36, 60, 90, 126, 168, and 216, using the structures reported in a previous study.^36^ ΔSCF final state CLBE values were calculated only for the central C atom of each flake, taken as a bulk-like reference (see Figure 5). For the remaining C atoms, and due to computational cost and convergence limitations, final state CLBEs were approximated via extrapolation of the initial state CLBE values (see Section S1 in the Supporting Information). These estimates were then compared to explicit final state results for the central C atoms (see Figure S4 in the Supporting Information). The extrapolated CLBEs slightly increase with flake size, whereas central C final state CLBE values remain nearly constant for both pristine and O-functionalized systems. This discrepancy is related to variations in the relaxation energy as a function of flake size. Larger flakes exhibit lower KS energies due to stabilization, leading to higher CLBEs. However, for central C atoms in Ti-based MXenes, ΔSCF final state CLBEs reveal that relaxation and stabilization effects compensate, resulting in an almost size independent CLBE.

**Figure 5:**
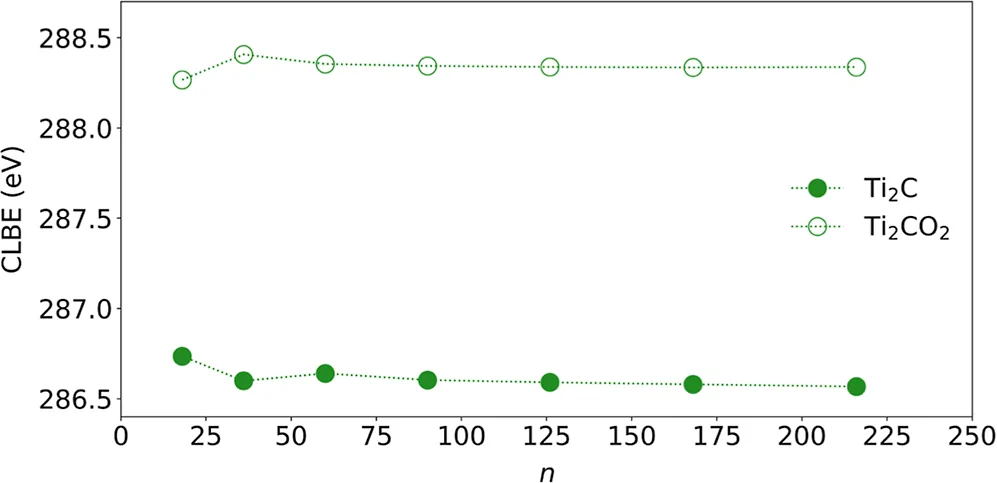
Central C­(1s) core-level binding energies (CLBE) vs flake size of (Ti_2_C)_*n*_ and (Ti_2_CO_2_)_*n*_ flakes with *n* = 18, 36, 60, 90, 126, 168, and 216. Filled and open circles correspond to pristine and O-functionalized MXenes, respectively.

The present results for (Ti_2_C)_*n*_ and (Ti_2_CO_2_)_*n*_ flakes indicate that the central C CLBEs remain essentially constant for larger flakes. Although a similar behavior may be expected for all Ti- and other metal-based systems, full verification is computationally prohibitive.

## 4 Conclusions

The present work reports an extensive study of the C­(1s) core-level binding energies (CLBEs) in (M_2_C)_18_ and (M_2_CO_2_)_18_ MXene flakes with M = Sc, Y, Ti, Zr, Hf, V, Nb, and Ta. All flakes exhibit ABC stacking and identical geometries. CLBEs were obtained using the ΔSCF approach, explicitly accounting for final state effects. For both pristine and O-functionalized flakes, it is found that the PBE functional reliably captures CLBE trends.

For all scrutinized MXene flakes, an increase in C­(1s) CLBEs is observed across periods, driven by decreasing atomic radii and increasing electronegativity of the transition metals, whereas trends along groups follow those of ionization potentials. The electronegative effect of the oxygen is clearly evident in O-functionalized flakes, leading to a systematic shift of CLBEs toward higher CLBEs and an increased energy range due to structural distortions. This shift is sufficiently large to enable differentiation between pristine and functionalized flakes and should be experimentally observable.

For a given (M_2_C)_18_ or (M_2_CO_2_)_18_ flake, the CLBEs of the different C atoms predicted from the ΔSCF approach appear to be quite close, indicating that conventional XPS measurements are expected to present a broad peak centered at the CLBE of the central C atoms (CLBE_C_).

The comparison to results derived from periodic models shows consistent trends in CLBE shifts, although quantitative differences remain. Furthermore, the central C­(1s) CLBEs in larger Ti-based MXenes are nearly independent of flake size, suggesting that the use of the (M_2_C)_18_/(M_2_CO_2_)_18_ models may be appropriate to estimate both position (CLBE_C_) and broadening (CLBE_Max_ – CLBE_Min_) in larger systems.

## Supplementary Material



## Notes

(Section S1) initial and final state effects; (Table S1) core-level binding energies (CLBE) and shifts (ΔCLBE) of the ESCA molecule with different basis sets and functionals; (Table S2) CLBE_Max_, CLBE_Min_, and CLBE_C_ for M_2_C and M_2_CO_2_ flakes; (Table S3) first ionization potential of the metals present on the studied MXenes; (Table S4) ΔCLBE for central C atoms of MXene flakes (ΔCLBE_Flake_) and periodic structures (ΔCLBE_Periodic_); (Figure S1) explanatory scheme of the definition and necessity of the ΔCLBE; (Figure S2) carbon–metal bond distances for M_2_C and M_2_CO_2_ flakes; (Figure S3) variation of the atomic charge on the central C and M atoms when functionalizing (M_2_C)_18_ flakes; (Figure S4) core-level binding energies (CLBE) vs flake size of Ti_2_C and Ti_2_CO_2_ flakes (PDF)
