# Supplementary material for: Disclosing Trends on C(1s) Core-Level Binding Energies of MXene Flakes: Metal, Size and Edge Effects
Source: J Phys Chem C Nanomater Interfaces. 2026 Aug 10;130(33):11693–700. doi: 10.1021/acs.jpcc.6c03476 (PMC13502393; doi:10.1021/acs.jpcc.6c03476)
Supplement: Supplementary file 1 [file jp6c03476_si_001.pdf]

Disclosing Trends on C(1s) Core-Level Binding Energies of MXene Flakes: Metal, Size and Edge Effects

Miquel Allés,[a] Sergi Vela,[b] Carmen Sousa,[a,*] Francesc Illas[a]

[a] *Departament de Ciència de Materials i Química Física & Institut de Química Teòrica i Computacional (IQTCUB), Universitat de Barcelona, c/Martí i Franquès 1-11, 08028 Barcelona, Spain*
[b] *Institut de Química Avançada de Catalunya (IQAC-CSIC), c/ Jordi Girona 18-26, 08028 Barcelona, Spain*

* Corresponding author e-mail: c.sousa@ub.edu

Table of contents

Section S1. Initial and final state effects

Table S1. Core-level binding energies (CLBE) and shifts (ΔCLBE) of the ESCA molecule with different basis sets and functionals.

Table S2. $CLBE_{Max}$, $CLBE_{Min}$, and $CLBE_{C}$ for M_2C and M_2CO_2 flakes.

Table S3. First ionization potential of the metals present on the studied MXenes.

Table S4. ΔCLBE for central C atoms of MXene flakes ($\Delta CLBE_{Flake}$) and periodic structures ($\Delta CLBE_{Periodic}$).

Figure S1. Explanatory scheme of the definition and necessity of the ΔCLBE.

Figure S2. Carbon-Metal bond distances for M_2C and M_2CO_2 flakes.

Figure S3. Variation of the atomic charge on the central C and M atoms when functionalizing $(M_2C)_{18}$ flakes.

Figure S4. Core-Level Binding Energies (CLBE) vs. flake size of Ti_2C and Ti_2CO_2 flakes.

References

Section S1. Initial and final state effects

As reported in the literature,[1-3] CLBEs are often dominated by initial state effects. Therefore, it is important to assess whether this also holds for the systems studied here. To this end, we compared the CLBEs obtained from the ΔSCF approach (which fully includes final state effects) with those estimated from Kohn-Sham (KS) orbital energies. It is worth recalling that, unlike in Hartree-Fock theory, where the negative of the orbital energy provides an estimate of the ionization potential of that electron, KS orbital energies do not formally provide an estimate of CLBE within the frozen orbital approximation. Nevertheless, previous studies have shown that KS orbital energies can yield reasonably accurate estimates of ΔCLBE values relative to a given reference.[4]

Computationally, the initial state estimate of the CLBEs by means of DFT calculations can be assessed through one ground state calculation, where the energy of the state with a core hole is computed from the density of the ground state, as explained in ref. 37 of the main text. In case one is only interested in CLBE shifts, the initial state CLBE of a targeted atom can be approached from the negative of the KS energy of that orbital, as shown in “Validation of Koopmans’ Theorem for Density Functional Theory Binding Energies”.[4] However, to compute the CLBE including final state effects an additional calculation is required, setting a positively charged system and, in the case of using the FHI-AIMS software, employing the *force_occupation_basis* tag, where one selects the specific electron that is removed from a given orbital. Using this last procedure, the CLBE is obtained as the difference in energy between the ground and core hole states. Let us point out that in the framework of Hartree-Fock theory, the initial state energy, as detailed above, corresponds exactly to the negative of the orbital energy, which follows from Koopman’s theorem (see refs. 22 and 23 of the main text).

The relevance of initial state effects is illustrated in Figure 1, which compares CLBEs predicted by the ΔSCF method (at the PBE level) and those derived from KS orbital energies. It should be noted that absolute CLBE values obtained from KS energies are underestimated, implying a positive relaxation energy upon ionization, which is not physically meaningful. This further confirms that KS energies cannot be used as a quantitative measure of initial state CLBEs. Despite this information, Figure 1 shows that, for the central C atoms of each MXene structure, a clear linear correlation emerges between final state CLBEs and the negative KS energies.

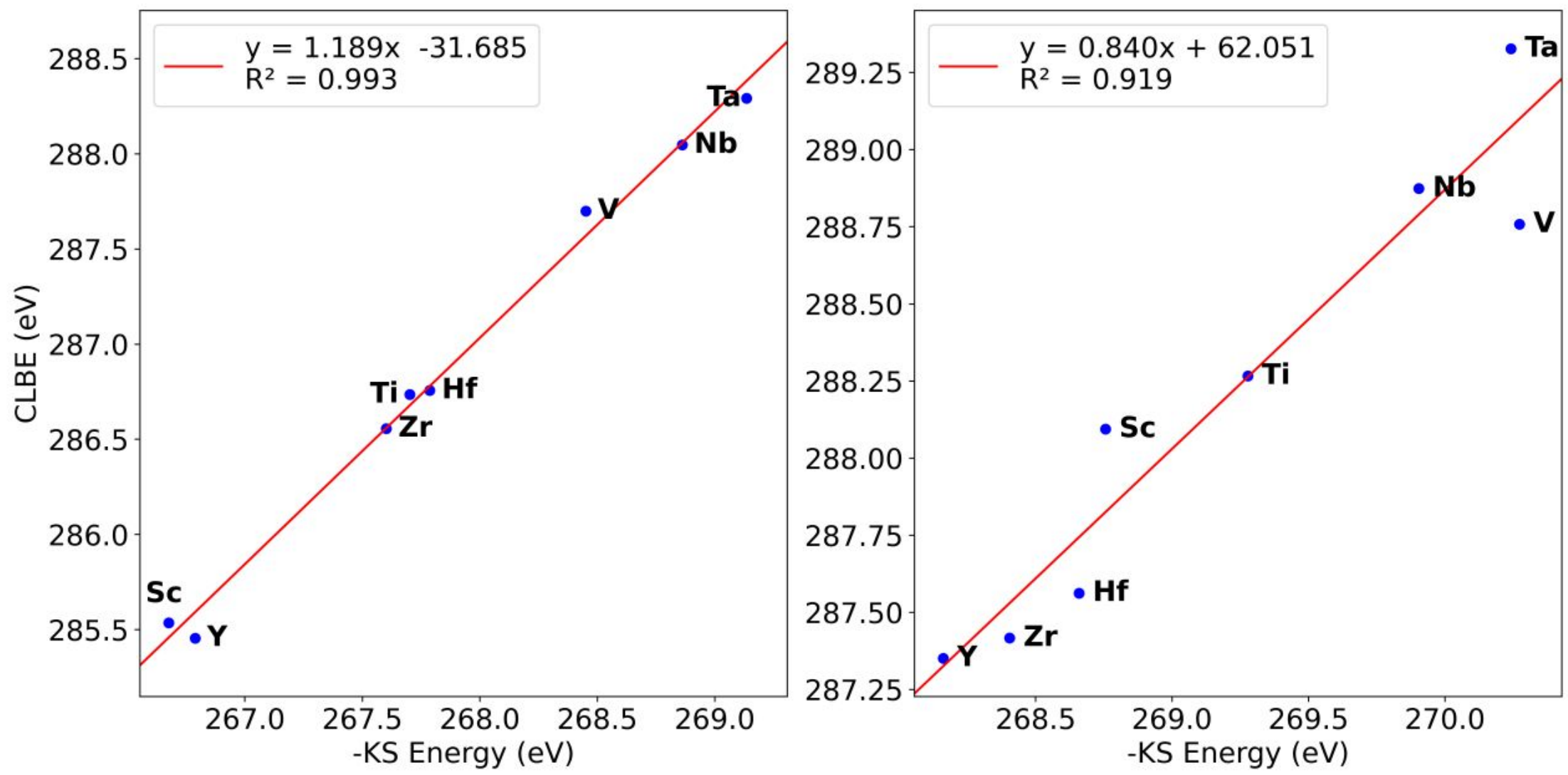

Figure 1. Linear regression between ΔSCF computed CLBEs and KS orbital energies for the central C atoms of $(M_2C)_{18}$ (left) and $(M_2CO_2)_{18}$ (right).

Very strong correlations are obtained for both pristine and O-terminated MXene flakes, with R^2 values of 0.993 and 0.919 for $(M_2C)_{18}$ and $(M_2CO_2)_{18}$ structures, respectively. These results indicate that differences in CLBEs across MXenes (*i.e.* the ΔCLBEs) are largely governed by initial state effects. Pristine flakes exhibit slightly stronger correlations than O-functionalized ones. In the latter, V-, Ta-, and Sc-based flakes appear as outliers. These deviations arise from differences in relaxation energies among the flakes and highlight the importance of explicitly accounting for final state effects to obtain accurate absolute CLBE values. For both pristine and O-functionalized MXenes, the similarity of the optimized structures leads to comparable relaxation energies. This is further illustrated in Figure 2, which compares CLBE-KS energy correlations for fully optimized and partially optimized $(M_2CO_2)_{18}$ structures. In the latter, the $(M_2C)_{18}$ structure is kept frozen while only the oxygen atoms are allowed to relax. For these partially optimized systems (M = Ti, Zr, Hf, V, Nb, and Ta), an excellent correlation is obtained (R^2=0.995) with a slope close to unity (1.008). This confirms that structural distortions induced by O adsorption influences both CLBE values and their relationship with initial state descriptors. Overall, these results support a strong correlation between initial and final state ΔCLBEs, linking ground state Kohn-Sham orbital energies with ΔSCF-derived CLBE values. This is consistent with the fundamental principle of XPS, where information about the ground state electronic structure is inferred from measurements

of the ionized system. Although structural distortions introduce variations in both CLBEs and relaxation energies, initial state effects remain the dominant contribution to ΔCLBE trends.

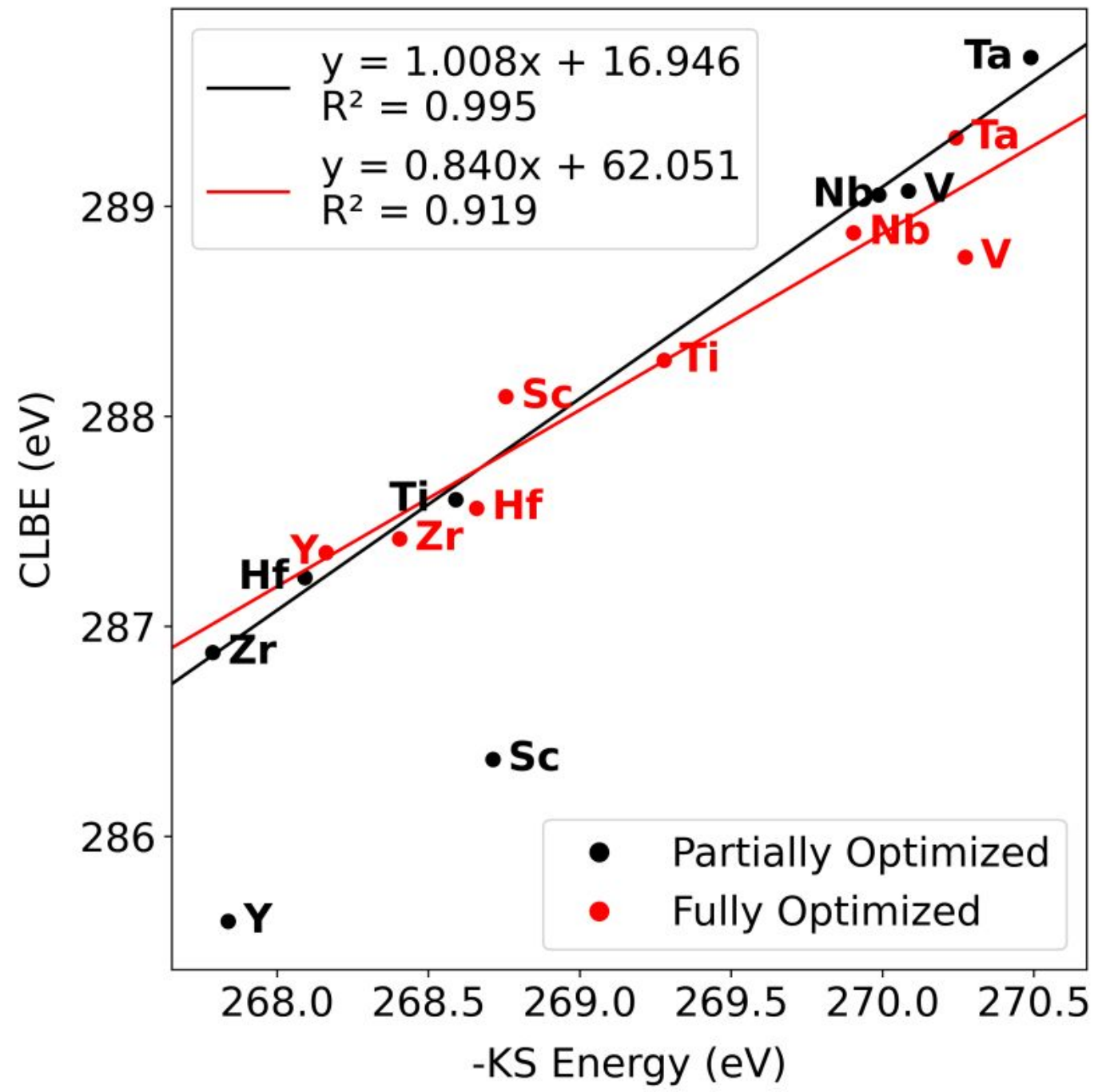

Figure 2. C(1s) Core-Level Binding Energy (CLBE) for the central C atom vs. Kohn-Sham energy for $(M_2CO_2)_{18}$ flakes. Fully optimized and partially optimized $(M_2CO_2)_{18}$ flakes are shown in red and black spheres, respectively. All values are given in eV.

In addition to the analysis of central C atoms, a more detailed investigation was performed to determine whether the same correlation holds across all C atoms within each flake, as illustrated for $(Sc_2C)_{18}$ in Figure 3. Pristine MXenes generally exhibit trends consistent with those observed for the central C atoms, with regression coefficients, R^2, ranging from 0.898 and 0.987. However, $(Y_2C)_{18}$ and $(Ti_2C)_{18}$ deviate significantly, with R^2 values of 0.457 and 0.302, respectively. In contrast, O-functionalized MXenes display more heterogenous behavior. Ti-, Zr-, Hf-, and Ta-based flakes retain strong correlations ($R^2 > 0.900$), whereas Sc- and Nb-based MXenes show intermediate correlations ($R^2 = 0.793$ and 0.700, respectively). Conversely, $(Y_2CO_2)_{18}$ and $(V_2CO_2)_{18}$ do not exhibit any clear trend, with correlation coefficients below 0.518. Overall, pristine MXene flakes exhibit more uniform structures and therefore stronger correlations, whereas O-functionalized flakes show greater structural distortions, leading to broader distributions of local environments and weaker correlations.

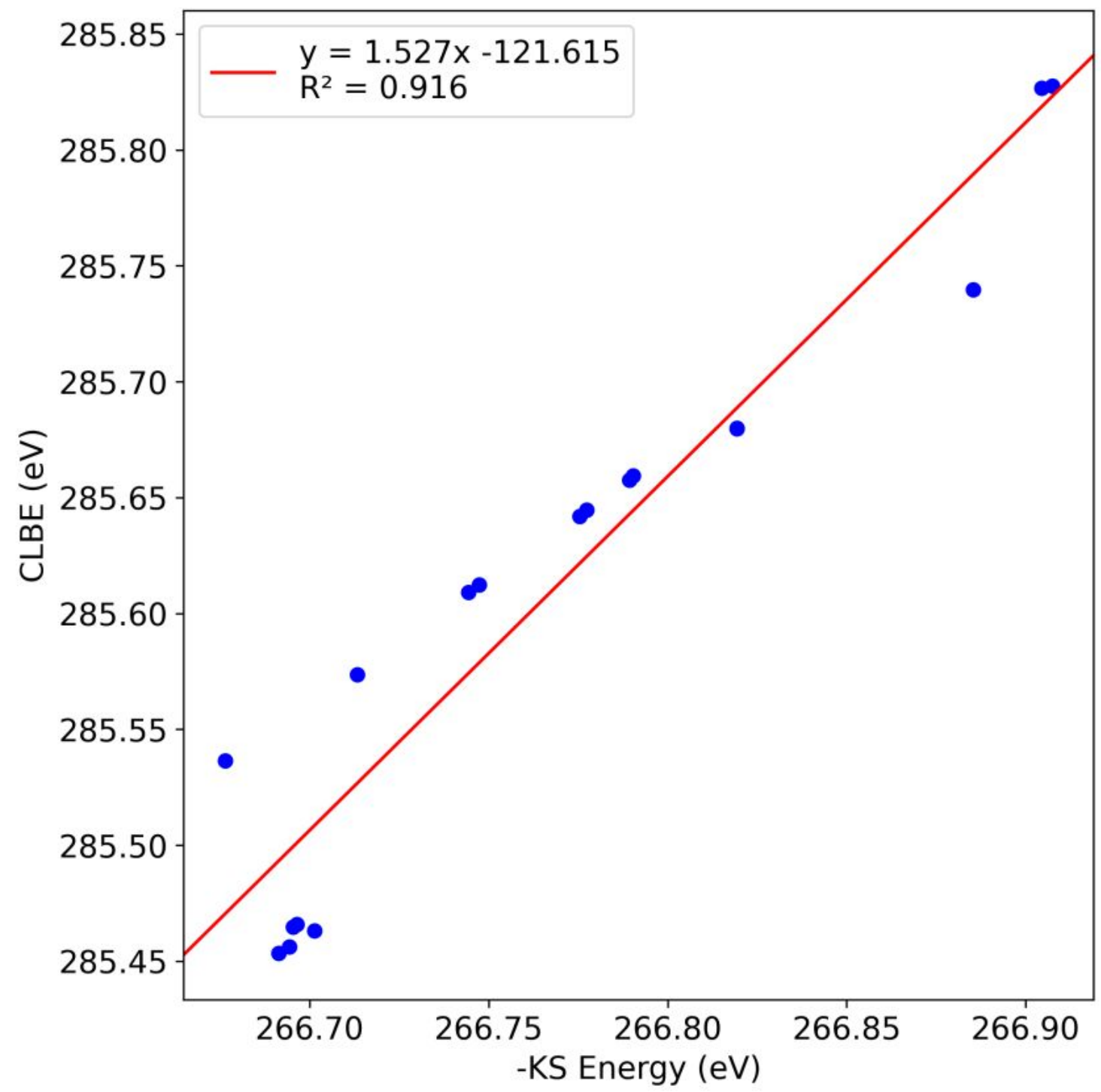

Figure 3. Linear regression between ΔSCF computed CLBE and initial state KS energies for all C atoms of $(Sc_2C)_{18}$.

Table S1. Carbon atoms Core-Level Binding Energies (CLBE) and shifts (ΔCLBE) of the ESCA molecule (ethyl trifluoroacetate) with different basis sets and functionals. All values are given in eV.[a]

PBE Tier 1 Light

	C_1		C_s	
	CLBE	ΔCLBE	CLBE	ΔCLBE
CF_3	299.33	6.98	299.34	6.92
C=O	296.28	3.92	296.26	3.84
O-CH_2	293.92	1.56	293.94	1.52
CH_3	292.36	-	292.42	-

PBE Tier 2 Light

	C_1		C_s	
	CLBE	ΔCLBE	CLBE	ΔCLBE
CF_3	299.13	7.05	299.14	6.98
C=O	295.97	3.88	295.95	3.79
O-CH_2	293.74	1.65	293.76	1.60
CH_3	292.09	-	292.16	-

PBE0 Tier 1 Light

	C_1		C_s	
	CLBE	ΔCLBE	CLBE	ΔCLBE
CF_3	300.13	7.50	300.14	7.44
C=O	297.11	4.48	297.08	4.39
O-CH_2	294.34	1.71	294.36	1.67
CH_3	292.63	-	292.69	-

PBE Tier 2 Tight

	C_1		C_s	
	CLBE	ΔCLBE	CLBE	ΔCLBE
CF_3	299.14	7.04	299.15	6.97
C=O	295.97	3.86	295.95	3.77
O-CH_2	293.75	1.65	293.77	1.59
CH_3	292.10	-	292.18	-

Experimental[42]

	ΔCLBE
CF_3	7.46
C=O	4.33
O-CH_2	1.72
CH_3	-

[a] PBE functional results are shown using Light and Tight accuracy with Tier 1 and Tier 2 basis sets. For PBE0 functional, Light accuracy with Tier 1 basis set results are presented. C_1 and C_s conformations of the ESCA molecule are displayed.

Table S2. Central ($CLBE_C$), minimum ($CLBE_{Min}$), and maximum ($CLBE_{Max}$) core-level binding energies for all M_2C and M_2CO_2 flakes. All values are given in eV.

	$CLBE_C$	$CLBE_{Min}$	$CLBE_{Max}$
**$(Sc_2C)_{18}$**	285.54	285.45	285.83
**$(Y_2C)_{18}$**	285.46	285.27	285.77
**$(Ti_2C)_{18}$**	286.74	286.11	286.74
**$(Zr_2C)_{18}$**	286.56	286.34	286.65
**$(Hf_2C)_{18}$**	286.76	286.50	286.83
**$(V_2C)_{18}$**	287.70	287.34	287.74
**$(Nb_2C)_{18}$**	288.05	287.51	288.05
**$(Ta_2C)_{18}$**	288.29	287.72	288.32
**$(Sc_2CO_2)_{18}$**	288.09	286.35	288.68
**$(Y_2CO_2)_{18}$**	287.35	285.72	287.76
**$(Ti_2CO_2)_{18}$**	288.27	287.97	289.29
**$(Zr_2CO_2)_{18}$**	287.42	287.12	288.50
**$(Hf_2CO_2)_{18}$**	287.56	287.22	288.53
**$(V_2CO_2)_{18}$**	288.76	288.47	289.81
**$(Nb_2CO_2)_{18}$**	288.87	288.60	289.31
**$(Ta_2CO_2)_{18}$**	289.33	288.91	290.11

Table S3. First ionization potential of the metals present on the studied MXenes. All values are given in eV.

**Sc**	**Ti**	**V**
6.56	6.83	6.75
**Y**	**Zr**	**Nb**
6.22	6.63	6.76
	**Hf**	**Ta**
	6.83	7.55

Table S4. Core-Level Binding Energy shifts (ΔCLBE) for the central C atom of MXene flakes ($\Delta CLBE_{Flake}$), MXene periodic structure ($\Delta CLBE_{Periodic}$).**Error! Bookmark not defined.** Ti-based MXenes were used as a reference. All values are given in eV.

	$\Delta CLBE_{Flake}$	$\Delta CLBE_{Periodic}$
Ti_2C	0.0	0.0
Zr_2C	-0.2	0.0
Hf_2C	0.0	-0.3
V_2C	1.0	0.8
Nb_2C	1.3	0.9
Ta_2C	1.6	1.1
Ti_2CO_2	0.0	0.0
Zr_2CO_2	-0.8	-0.5
Hf_2CO_2	-0.7	-1.1
V_2CO_2	0.5	1.0
Nb_2CO_2	0.6	1.3
Ta_2CO_2	1.1	1.6

Figure S1. Explanatory scheme of the definition and necessity of the ΔCLBE.

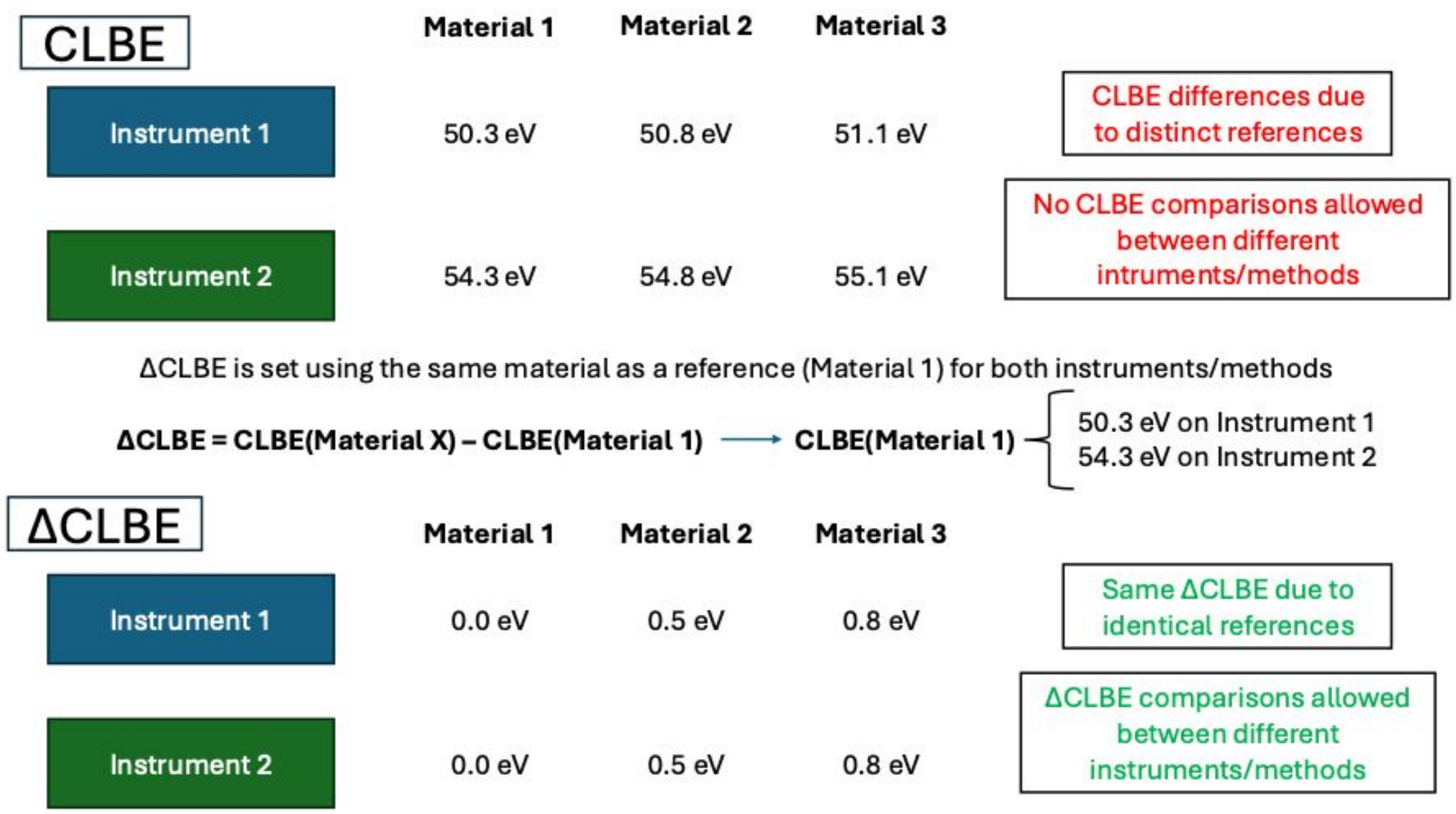

Figure S2. Carbon-Metal bond distances for $(M_2C)_{18}$ and $(M_2CO_2)_{18}$ flakes. $(M_2C)_{18}$ and $(M_2CO_2)_{18}$ flakes are represented with full and empty spheres, respectively. All values are given in Å.

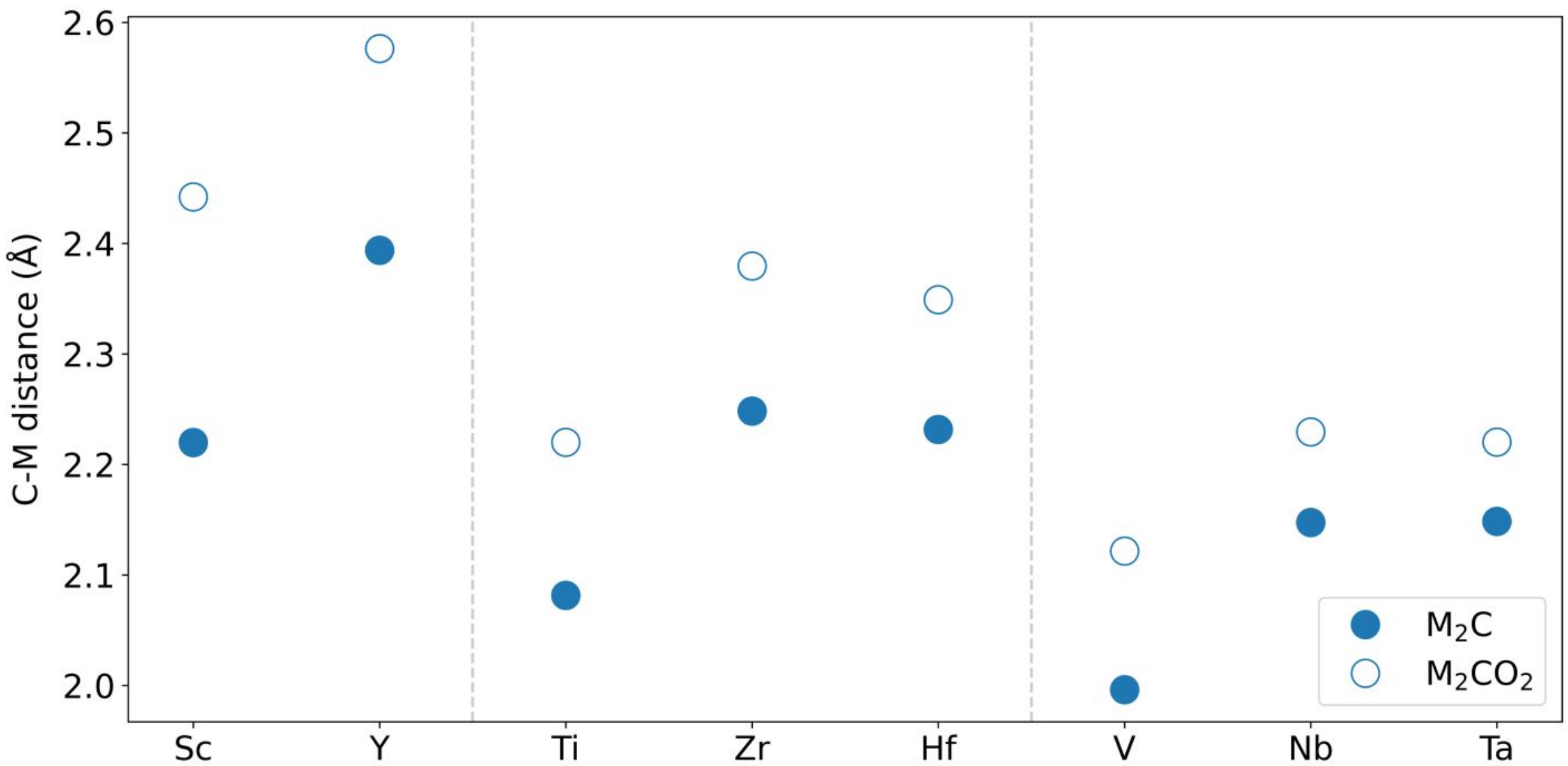

Figure S3. Variation of the atomic charge on the central C and M atoms when functionalizing with O the $(M_2C)_{18}$ flakes. Carbon and metal atoms are shown in full and empty spheres, respectively. All values are given in electrons.

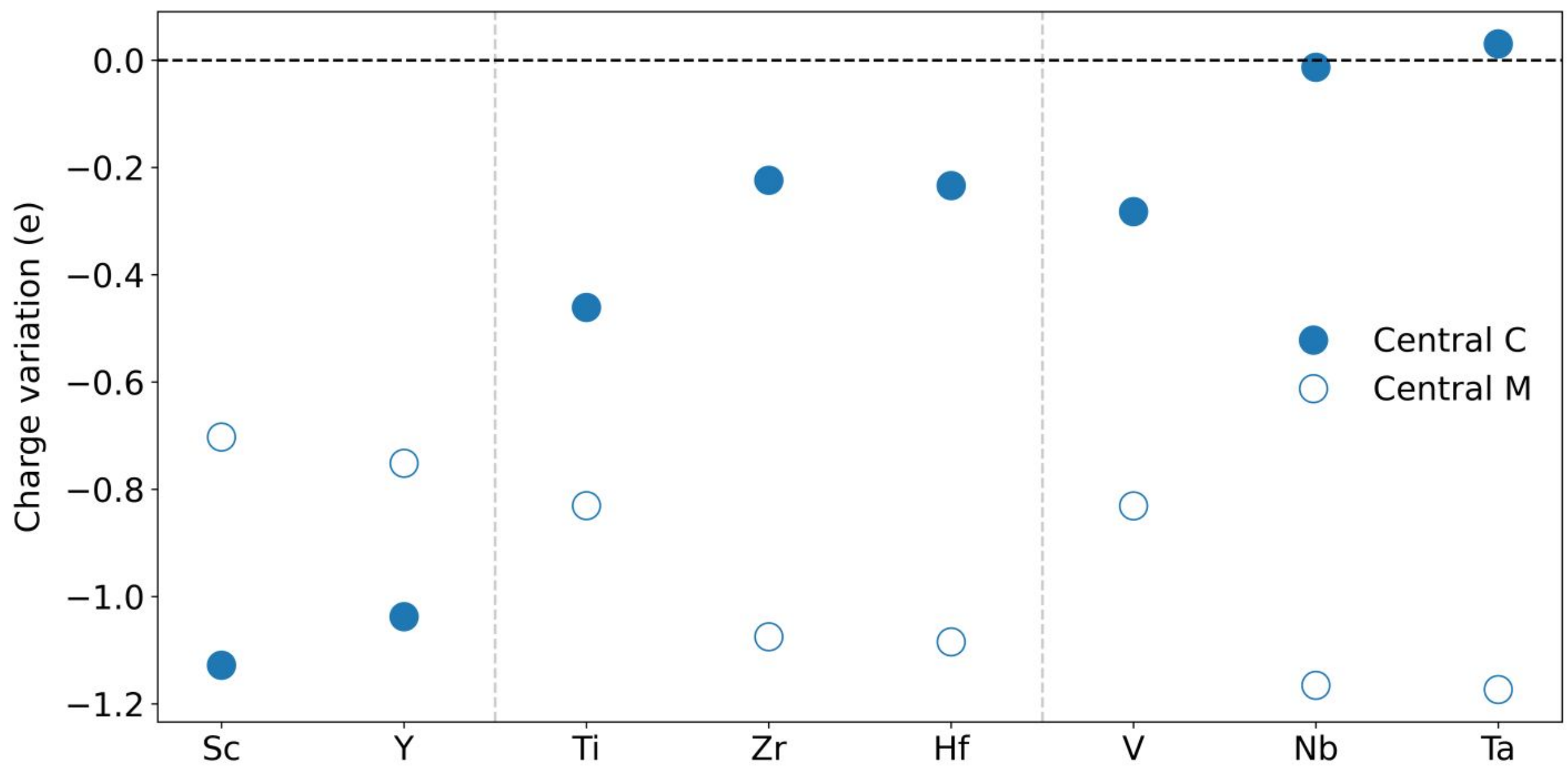

Figure S4. Core-Level Binding Energies (CLBE) vs. flake size of $(Ti_2C)_n$ and $(Ti_2CO_2)_n$ flakes. The approximated minimum (Approx. $CLBE_{Min}$) and maximum (Approx. $CLBE_{Max}$) values for each flake are shown in blue and orange, respectively, while the energy of the central carbon atom (Approx. $CLBE_C$) is shown in black. Green values correspond to $CLBE_C$ of MXene flakes with n = 18, 36, 60, 90, 126, 168 and 216. Filled and open circles correspond to $(M_2C)_{18}$ and $(M_2CO_2)_{18}$ MXenes, respectively.

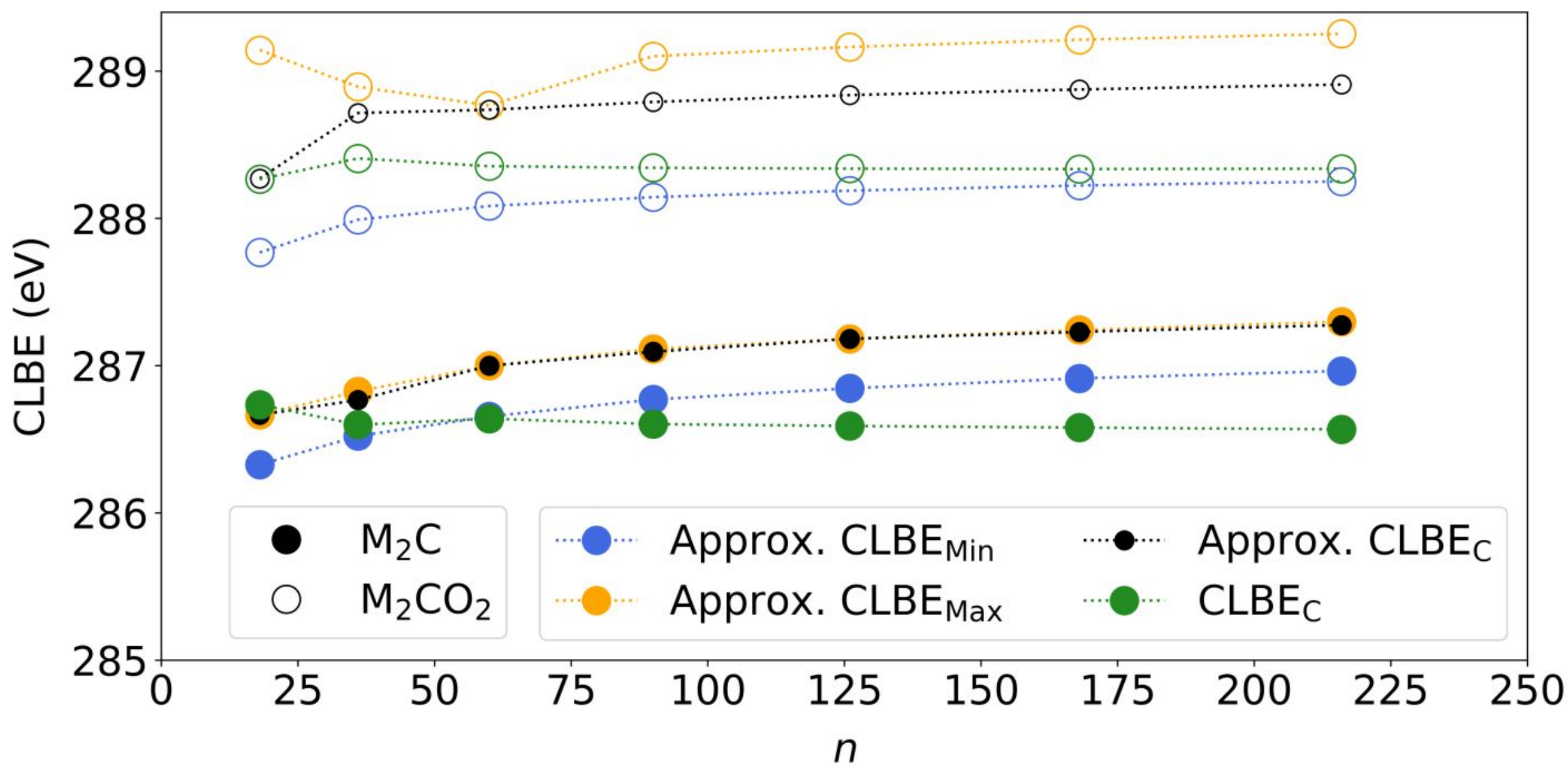

References

1 P.S. Bagus, F. Illas, G. Pacchioni and F. Parmigiani, *J. Electr. Spectrosc. Relat. Phenom*., 1999, **100**, 215–223.

2 P. S. Bagus, E. S. Ilton and C. J. Nelin, *Surf. Sci. Rep*, 2013, **68**, 273–304.

3 P. S. Bagus, E. Ilton and C. J. Nelin, *Catal. Letters*., 2018, **148**, 1785–1802.

4 N. Pueyo Bellafont, F. Illas, P. S. Bagus, *Phys. Chem. Chem. Phys.*, 2015, **17**, 4015-4019.
